# Should NIH restrict the use of grant funds for publication costs? An analysis of public comments

**DOI:** 10.1093/haschl/qxag163

**Published:** 2026-11-05

**Authors:** Robert M Kaplan

**Affiliations:** Clinical Excellence Research Center, Stanford University School of Medicine, Palo Alto, CA 94304, United States

**Keywords:** academic publishing, scholarship, conflicts of interest, equity

## Abstract

Rapid growth in open-access publishing has reshaped the economics of scientific communication. In response to concerns about the cost and process of commercial publishing, the National Institutes of Health (NIH) issued a request for information (RFI) in 2025. This study presents an independent analysis of the responses of all 914 responses from individuals and organizations, comprising more than 1300 pages. Narrative comments were categorized into themes and examined in relation to policy options. Response patterns varied by topic. Critical comments were most frequent regarding article processing charges (APCs) (445/914, 48.7%), followed by concerns about commercial publishing (210/914, 23.0%), unpaid academic labor (96/914, 10.5%), and peer review (50/914, 5.5%). Supportive comments were most common for peer review (84/914, 9.2%) and rare for APCs (0.2%), unpaid labor (1.2%), and commercial publishing (2.1%). The public comments highlight tensions in the current publishing system. Respondents endorsed the central role of peer review while expressing concern about APCs, market concentration, and reliance on unpaid labor. Differences between individual and organizational responses suggest competing stakeholder incentives. Policies focused narrowly on cost containment, such as APC caps, may fail to address structural issues and risk unintended consequences.

Scholarly publication in peer-reviewed journals remains central to academic advancement and the dissemination of scientific knowledge. Yet the contemporary publishing ecosystem faces mounting structural pressures that have raised questions about sustainability, equity, and the future of peer review.^1-3^

On July 30, 2025, the director of the National Institutes of Health (NIH) released a request for information (RFI) on maximizing research funds by limiting allowable publishing costs (NOT-OD-205-138). To increase productivity under budgetary constraints, NIH expressed concerns about paying unreasonably high article processing charges (APCs), fees authors pay to publish open-access articles. The RFI emphasized opportunity costs. A recent report from the Government Accountability Office found that the estimated publication costs for 7 scientific agencies will exceed $1 billion per year between 2026 and 2030. On a fixed budget, these high publication costs will result in the fewer funds available to support original research studies.^4^ This paper reviews the developing crisis in academic publishing and presents an analysis of responses to the NIH RFI.

 

### The crisis in academic publishing

Historically, scholarly journals were largely managed by academic institutions and professional societies.^5^ Over time, however, the logistical demands associated with editorial management, printing, distribution, and archiving increased substantially. These operational challenges gradually shifted responsibility for publication to large commercial publishing organizations, transforming scholarly communication into a highly consolidated industry.^6-9^

Today, academic publishing represents a major commercial enterprise.^1^ Although data are hard to find, it has been reported that academic publishing is one of the world's most lucrative industries in terms of profitability, with profit margins approaching 40%^10^—considerably higher than 2023 profits for Netflix (10.6%) or Amazon (6.25%). Even Apple, the world's second-largest company, had a lower margin, at 28.4%.^11^ Fossil fuel giants are far less profitable than academic publishers: In 2023, Shell Oil reported a high profit margin of 7.27%. Meanwhile, Elsevier reported a 38.1 profit margin in 2025.^12^

For decades, publishing industry revenues were supported primarily by library subscriptions funded indirectly through university budgets and overhead on research grants. As library purchasing power has stagnated, publishers have increasingly shifted costs toward authors through APCs. This transition coincided with the expansion of open-access policies, creating new financial dynamics that affect investigators, institutions, and funders.

Although peer review remains a cornerstone of scientific credibility, concerns about its sustainability have intensified. Journalistic coverage often treats “peer-reviewed” research as inherently authoritative. Yet scholars,^13-15^ librarians,^16-18^ and editors have documented limitations in the current system.^5-9^ These concerns reflect both operational pressures and evolving expectations surrounding transparency, reproducibility, and accessibility.

### Editorial perspectives

Journal editors now face common operational challenges. In particular, securing qualified reviewers has become increasingly difficult. In earlier decades, invitations to 5 established experts typically yielded 2 or 3 completed reviews. Contemporary editors now send 15-25 invitations to obtain the same number of responses.^19,20^ The expansion of journal titles, combined with the lack of compensation for reviewers,^21^ has substantially increased demands on a limited pool of completely overwhelmed scholars.^22,23^

Variability in review quality, reflecting time constraints and competing professional responsibilities, has also raised concerns.^20^

### Open-access publishing

In principle, taxpayer-supported research should be a public good that is openly available to everyone.^20^ As outlined in the Budapest Initiative,^24^ academic open-access publishing is a model of scholarly communication in which research articles are made freely available online without subscription or paywall barriers. The stated goal is to increase access to scientific knowledge for researchers, clinicians, policymakers, and the general public. Open access can be achieved through several mechanisms, including publication in fully open-access journals, publication of individual open-access articles in traditional subscription journals, or deposition of manuscripts in public repositories. Advocates argue that open access accelerates scientific progress, increases the visibility and impact of research, and ensures that findings derived from publicly funded research are available to all. However, the model has also generated controversy because publication costs are often shifted from readers to authors through APCs.

### APCs and access

Article processing charges have grown substantially. Because of the high costs, many scholars are now unable to afford to publish their work in high-visibility journals.

The NIH estimated that the mean APC in 2025 was $2176.84 (range $300.00 to about $12 000). Butler and colleagues estimated that authors paid the 6 largest publishers approximately $1.6 billion in APCs between 2015 and 2018.^9^ These costs may be particularly burdensome for early-career investigators and researchers in low- and middle-income settings. In many cases, APCs are paid for with research grants or institutional funds, diverting resources that might otherwise support original research.

The author-pays model also raises structural questions. Authors frequently provide manuscripts without compensation, and reviewers contribute unpaid labor that improves the work. Yet commercial publishers collect APC revenue while relying on volunteer contributions. Universities occasionally subsidize publication fees, but institutional funds are typically limited. As APC expenditures increase, institutions face trade-offs that affect library collections, faculty support, and student services. Brembs and colleagues describe a cycle in which institutions increasingly pay subscription or APC fees while faculty simultaneously provide unpaid editorial and review labor.^14^ Meanwhile, commercial publisher profits continue to flourish.

In addition to structural concerns, there are ethical questions about a publishing model that rewards commercial publishers for increasing the number of articles they publish, regardless of their quality.

### From subscription to author-pays models

Article processing charges emerged partly in response to the limitations of subscription-based publishing. Under the subscription model, universities and individuals paid for access to journals, often at substantial cost. Although this approach supported publication infrastructure, it restricted access for readers without subscriptions. Open-access policies sought to improve availability, but the resulting shift toward APCs has redistributed rather than eliminated financial burdens.

### Request for information

A RFI is a request for information. It is a formal mechanism for the US federal government to request public statements. On January 8, 2025, NIH announced its intent to create new policies that would limit the use of federal funds to cover publication costs. Before implementing the policy, originally planned for January 1, 2026, the NIH provided the opportunity for members of the research community to offer public comments. In particular, they offered 5 options:

Option 1: Disallow all publication costs.

Option 2: Set a limit on allowable costs per publication.

Option 3: Set a limit on allowable costs per publication and allow a higher amount to be paid when peer reviewers are compensated.

Option 4: Set a limit on the total amount of an award that can be spent on publication costs.

Option 5: Set a limit on both the per-publication cost and the total amount of an award that can be spent on publications.

This paper summarizes responses to the RFI and provides commentary on future directions

## Method

The NIH Request for Public Comment on Maximizing Research Funds by Limiting Allowable Publishing Costs provoked an unusually broad response. Responses were received from 914 individuals or organizations, producing a compilation exceeding 1300 single-spaced pages. ChatGPT was used to place narrative comments into broad categories.

### Cross validation

A 5% random sample (*n* = 46) of the 914 public comments was human coded by the author using a structured codebook capturing positions on APCs, proposed caps on publishing costs, peer review practices, equity considerations, administrative burden, and support for alternative publishing models. Inter-rater reliability between the artificial intelligence (AI) and human coders was high, with an overall agreement of approximately 90% (Cohen's kappa ∼0.8), indicating substantial agreement across coded domains.

## Results

A total of 827 individual and 87 organizational responses were analyzed (total = 914). The organizations included universities, libraries, professional societies, and research institutions. Selected representative comments are shown in Table 1. The comments addressed multiple themes related to the economics and structure of academic publishing.

**Table 1.** Selected illustrative comments on APC pricing, commercial publisher power, and market structure.

Commenter	Affiliation/role	Illustrative quote	Page
Gloria Greene	University of Alabama in Huntsville; organizational response	“Strict constraints on publication cost recovery limit researchers’ ability to disseminate high-quality, peer-reviewed work … Rather than imposing blanket cost ceilings, NIH should continue to allow reasonable publication costs.”	30
Mary Cushman	University of Vermont; investigator	“Most investigators, myself included, will not pay egregious fees unless the journal is of such high quality that this will (1) provide the best dissemination of their research, and (2) publish in a journal with the esteem needed to impress reviewers.”	32
Gail Bishop	University of Iowa; investigator	“Fees … have increased substantially over the past 10-15 years, with the huge explosion that OA policy has stimulated in ‘for-profit’ publishers.”	33
Anonymous commenter	Nonprofit research organization; investigator	“Most APCs that I look up when considering journal choice are at least $2800. Small labs cannot afford to publish in the most expensive journals.”	35
Dmitriy Sheyn	Cedars-Sinai Health Sciences University; investigator	“If the policy will force the publishers to reduce APCs, that's great, but if the policy will only restrict the researchers and will force [them] to come up with alternative funding for publishing the work, it will be counter productive.”	36
Alex M	Academic institution; organizational official	“APCs … have resulted in the proliferation of substandard/questionable journals … and have further consolidated the market in the big publishers, as they are the only ones with the economies of scale required for Gold OA-based publishing.”	37
Anonymous commenter	Academic institution; investigator	“While I share NIH's concern about the rising costs of scholarly publishing—particularly the exorbitant APCs charged by some for-profit journals—I believe that simply capping allowable costs on NIH grants is a blunt instrument.”	40
Kristin Riekert	Investigator	“In my field, publishing costs are usually >3K and rising, and I cannot remember the last time I saw one <$1500.”	47
Gary L. Westbrook, MD	Vollum Institute; investigator	“Commercial publishers, particularly Cell Press and Springer are falsely elevating the costs of manuscript publishing just to make a profit. The actual costs are in the 2-3K range not the 12K charged by e.g. Nature or Cell.”	49
Cathy Stein, PhD	Case Western Reserve University; investigator	“The minimum that I have spent recently on a paper publication cost is $3000. Some journals charge much more. What's worse, is journals charge even more for ‘open access’ … That's absurd.”	53
Vanessa Brown	Investigator	“APCs are generally higher for higher impact factor journals.”	55
Catherine Murphy	University of Illinois Urbana-Champaign; investigator	“Nature is up to $12 000 or so for an open-access paper … the charge to authors now bears little resemblance to the actual costs of publishing.”	64
Cynthia Monaco	Investigator	“With the move to open access publishing, the cost of publications has increased substantially. My publication costs for my last 4 publications submitted this year have averaged over $4000.”	65

### Overall themes

The responses clustered around 5 themes: (1) high APCs, (2) commercialization of academic publishing, (3) exploitation of peer reviewers, (4) the importance of peer review, and (5) equity. There was substantial heterogeneity in how respondents evaluated different thematic categories of the NIH RFI across (χ^2^(9) = 2333.16, *P* < 0.001). The distribution of response categories varied substantially by topic. A summary of response frequencies is given in Table 2.

**Table 2.** Summary of coded response frequencies.

Topic	Count	Percent
Importance of peer review		
Positive/supportive	84	9.19%
Critical/concern raised	50	5.47%
Neutral	780	85.34%
No relevant comment	0	0.00%
Concerns about APCs		
Positive/supportive	2	0.22%
Critical/concern raised	445	48.69%
Neutral	467	51.09%
No relevant comment	0	0.00%
Unpaid academic labor		
Positive/supportive	11	1.20%
Critical/concern raised	96	10.50%
Neutral	807	88.29%
No relevant comment	0	0.00%
Concerns about commercial publishing		
Positive/supportive	19	2.08%
Critical/concern raised	210	22.98%
Neutral	239	26.15%
No relevant comment	446	48.80%

Count is the frequency of each response. The denominator for the calculation of each percentage is 914 (representing the number of responses to the RFI).

#### Article processing charges

Critical comments were most common for APCs (see representative comments in Appendix Table S1). Nearly half of respondents (445/914 or 48.7%) explicitly criticized APCs, while an additional 51.1% (467/914) discussed APCs in neutral or descriptive terms. Very few respondents expressed support for APC levels or trends. These comments frequently described APC growth as a systemic issue tied to journal prestige, market concentration, and academic incentives rather than a simple administrative burden.

#### Commercialization

Concerns about commercial publishing were also common. Approximately 23.0% (210/914) of respondents directly criticized commercial publishers, while 261 of 914 respondents (26.1%) discussed commercial publishing in neutral terms. Nearly half of the comments (446/914; 48.8%) did not explicitly address commercial publishers. When raised, critiques focused on pricing power, market consolidation, and the misalignment between publicly funded research and privately captured publishing revenue.

#### Peer reviewer exploitation

A smaller but notable subset of respondents emphasized reliance on unpaid academic labor. About 10.5% (96/914) of comments explicitly highlighted unpaid peer review or editorial work as a concern. These comments frequently noted that reviewers and editors contribute substantial uncompensated labor while profitable publishers collect APC revenue. Many additional comments discussed peer review indirectly without explicitly framing unpaid labor as a concern (selected comments are given in Appendix Table S2).

#### Importance of peer review

Explicit positive statements emphasizing the importance of peer review appeared in 9.2% (84/914) of responses. These comments often argued that any reforms to publishing costs should preserve rigorous peer review and editorial oversight (see Appendix Table S3).

### Equity implications

High APCs may also create inequities. Early-career investigators, small laboratories, and researchers at less-resourced institutions may face barriers to publishing in high-impact journals. Multiple respondents noted that publication fees can be paid from personal funds or departmental budgets when grant funding is insufficient, potentially disadvantaging investigators without such resources.

Overall, positive or supportive comments were most common for peer review (84/914, 9.2%) and were uncommon for APCs (2/914, 0.2%), unpaid academic labor (11/914, 1.2%), and commercial publishing (19/914, 2.1%).

### Differences between individual and organizational respondents

Important differences emerged between individual and organizational respondents. Organizations were more likely to emphasize structural features of the publishing system, whereas individuals more often highlighted practical and financial burdens.

Organizations were more than twice as likely as individuals to emphasize the importance of peer review (18.4% vs 8.2%; χ^2^ [1 df] = 9.74, *P* < 0.002). These comments frequently stressed the need to preserve editorial quality and maintain rigorous peer review processes in any reforms to publication costs.

Individual respondents were more likely than organizational respondents to highlight reliance on uncompensated peer review and editorial labor (11.2% vs 3.4%; χ^2^ [1 df] = 4.94, *P* < 0.05). These comments often described peer review as essential but increasingly difficult to sustain without recognition or compensation. Concerns about APCs were common in both groups, though slightly more frequent among organizations (54.0%) than individuals (48.1%) (χ^2^NS). Both groups described APC growth as problematic, but organizations more often framed APC inflation as a structural market issue, whereas individuals frequently emphasized the direct financial burden on investigators and research teams.

Criticism of commercial publishers also appeared more frequently among organizations (28.7%) than individuals (22.4%), although the difference was not statistically significant. Organizational responses often discussed market concentration, pricing power, and systemic incentives, while individual responses focused more on publication costs and journal selection constraints.

## Discussion

The 914 responses, spanning nearly 1300 single-spaced pages, addressed a wide range of issues related to NIH's request for information on limiting allowable publication costs. Respondents differed sharply on whether limiting allowable publication costs on NIH grants is an effective or equitable mechanism for achieving this goal. Many commenters endorsed improving access but cautioned that blunt cost caps could create unintended consequences affecting research dissemination, equity, and the functioning of peer review.

Beyond consistent endorsement of peer review, 4 interrelated concerns dominated the responses: rising APCs, reliance on an unpaid peer-review workforce, increasing concentration of power among commercial publishers, and equity concerns. Respondents frequently described substantial increases in publication costs over the past decade and noted that APCs vary widely across journals, often appearing only loosely related to underlying publication expenses. Many commenters argued that pricing is driven less by production costs than by journal prestige, audience reach, and career incentives, allowing high-impact commercial journals to charge substantially higher fees than nonprofit or society journals. In this environment, APC inflation was often characterized as a structural feature of the current publishing ecosystem rather than a simple administrative inconvenience.

Concern about dependence of scholarly publishing on uncompensated academic labor is consistent with a variety of previously published commentaries.^1-3^ Respondents consistently emphasized that peer review remains central to scientific credibility but is increasingly strained.^25,26^ Consistent with prior critiques,^27-29^ reviewers and editors provide substantial unpaid labor,^30^ while publishers collect APC revenue. Many commenters argued that this arrangement effectively subsidizes the publishing system with publicly funded academic work.^31^ At the same time, respondents stressed that peer review is becoming more resource-intensive, citing increased submission volume, the growth of journal titles, and concerns about paper mills and AI-assisted misconduct.

Although there was broad agreement about the importance of peer review, views diverged on whether reviewers should be compensated. Proponents argued that peer review represents skilled labor with opportunity costs and that compensation might improve timeliness, accountability, and equity for investigators with heavy clinical, teaching, or administrative responsibilities. Opponents—including many editors, scholarly societies, and nonprofit publishers—warned that financial incentives could undermine reciprocal norms, distort reviewer motivation, and introduce substantial administrative overhead. Some also expressed concern that large commercial publishers could outbid nonprofit journals for experienced reviewers, potentially creating a 2-tier system in which elite reviewers gravitate toward higher-paying journals.

A different perspective was offered by Marcum and Turbes^32^ who also reviewed selected responses to the NIH RFI and concluded that there is persistent confusion regarding the costs, scope, and implications of the NIH Public Access Policy. As they note, the requirement that NIH-funded research articles be made publicly accessible through PubMed Central does not obligate authors to publish in open-access journals or to pay APCs. Rather, the policy permits multiple compliance pathways, including deposit of the peer-reviewed manuscript following publication in a traditional subscription journal.

Marcum and Turbes further argue that critics frequently conflate public access mandates with open-access publishing models, thereby overstating the financial burden on researchers and institutions. In practice, the policy is designed to expand public access to federally funded research without dictating publication venue or requiring payment to publishers. Many journals already support compliant deposition workflows, often at no additional cost.

However, this framing addresses only part of the problem. While the policy itself may be modest and flexible, it operates within a broader publishing ecosystem in which commercial incentives, pricing practices, and reliance on unpaid academic labor continue to drive costs upward. To the extent that Marcum and Turbes emphasize communication and education over structural reform, their approach risks understating these underlying dynamics. Improving understanding of the policy may reduce confusion, but it does little to address the more fundamental question of whether the current system for disseminating publicly funded research is economically efficient or aligned with the public interest.

Concerns about market structure also featured prominently in this review and in other published evaluations.^33^ Many respondents distinguished between commercial publishers and nonprofit or society journals, arguing that APC-based models may disproportionately benefit large publishers with economies of scale. Commenters warned that policies limiting allowable publication costs could unintentionally accelerate consolidation by favoring publishers able to operate efficiently at scale while threatening smaller society and diamond open-access journals that often provide more intensive editorial oversight. These concerns were closely linked to equity issues.^34^ Respondents noted that publication-cost restrictions could shift financial burdens to investigators or institutions, increase out-of-pocket expenses, and reduce access to high-visibility journals. Early-career investigators were frequently identified as particularly vulnerable to policies that constrain dissemination options at critical career stages.

Taken together, the comments highlight a broader tension between the goals of public accessibility, affordability, and sustainability. While there was strong support for ensuring that NIH-funded research is publicly available, respondents emphasized that publication costs cannot be considered in isolation from the structure of the publishing market or the labor supporting peer review. Article processing charge-driven models may create misalignment between public investment in research and the costs of dissemination, while reliance on uncompensated academic labor raises questions about long-term sustainability. Policymakers considering limits on publication costs therefore face the challenge of balancing affordability with maintaining high-quality peer review, equitable access to publication venues, and a diverse scholarly publishing ecosystem.

### Publicly funded research as a public good

Elsewhere, I have argued that publicly funded research is intended to function as a public good.^20^ To achieve that status, research publications must meet 2 criteria. First, publications must be nonrivalrous, meaning one person's use does not diminish their value to others. Further, publications must be nonexcludable. They should be accessible to all without barriers. The current academic publishing system often undermines these properties by restricting access through paywalls and shifting costs to authors via high APCs. This has effectively commercialized taxpayer-funded knowledge within a highly profitable global industry that depends heavily on unpaid academic labor. Mechanisms such as “read-and-publish” agreements expand access only within paying institutions while reinforcing inequities and consolidating market power among large publishers. Although open-access initiatives, such as nonprofit platforms and institutional publishing models, offer more equitable options, they still face challenges with sustainability, incentives, and visibility.

Meaningful reform will require coordinated efforts by funders, institutions, and scholarly societies to realign dissemination practices with the core principle that publicly funded science should remain openly accessible to all.

### Limitations

This analysis must be interpreted in light of several important limitations. First, individuals and organizations who respond to a request for comment cannot be considered a representative sample of research investigators. Although 914 individuals or organizations responded, the NIH research enterprise is much larger. Each year, NIH funds nearly 60 000 grants to over 300 000 researchers in more than 2500 institutions.

A second important limitation is that the coding of responses was primarily achieved using AI. This was necessary because of the demands required to read and consistently classify more than 1300 single-spaced pages. It is important to recognize that AI, like humans, makes classification errors. It is, however, gratifying that the independent review of 5% of the comments is largely consistent with the AI coding. All of the original comments are in the public domain, and additional analyses are encouraged.

### Alternative publishing systems

Most of the discussion in this paper has been focused on the problem of high-cost APCs. However, several other models of open-access publishing have emerged. Hybrid Open Access journals operate primarily on a subscription basis but allow individual articles to be made open access if authors pay an APC. Green Open Access permits authors to publish in traditional journals while also depositing a version of their manuscript in a public repository, often after an embargo period. The Diamond (or Platinum) Open Access makes articles freely available to readers without charging authors publication fees, with costs supported by universities, scholarly societies, foundations, or government agencies. Each model seeks to improve access to research but differs in how publication costs are distributed among authors, readers, institutions, and funders.

The problem with each of these models is that they remain dependent on high-cost, often for-profit publishers. Rather than relying so heavily on large commercial publishers, universities and scholarly societies are well positioned to manage journals themselves. Rigorous peer review should remain central, but the individuals who perform it deserve formal recognition. Systematic evaluation of review quality could allow these contributions to count meaningfully in promotion and tenure decisions.

## Conclusions

Taken together, the public comments convey a clear message: The community shares NIH's concern about rising publication costs, but is deeply skeptical of restrictive caps on allowable costs. If implemented without attention to peer review and publishing practices, capping publication costs may not achieve the intended goals.

Peer review is widely seen as fragile, essential, and under strain. While there is interest in better recognizing reviewer labor, most respondents caution that financial compensation may introduce more problems than it solves, particularly if imposed through publication-cost policy rather than addressed in the context of high-quality science and academic values.

To address these concerns, alternative systems should be considered. One model^19^ would eliminate outsourcing research dissemination to multinational corporations and return to models run by universities and scholarly societies. Peer review is essential, but reviewers should be recognized and credited for their work. The quality of reviews could be rated and used as evidence to justify promotion. Universities and funders could invest in infrastructure that supports open publishing: hosting platforms, editorial staff, and long-term digital archiving. The NIH cap on APCs is a good start, but a unified academic community could achieve much more. Institutions now pay substantially different rates to obtain access to the same package of journals.^35^ Using the NIH caps as a guide, a consortium of institutions could use their monopsony power to set the prices they will allow.

The billions of dollars currently spent on APCs and subscriptions could instead underwrite these services. Articles could be published as open-access PDFs with searchable metadata, persistent identifiers, and copyright retained by the authors or their institutions.

The commercial publishing model is no longer fit for purpose. It exploits publicly funded science, depends on unpaid labor, and restricts access to knowledge that should belong to everyone. The NIH's recent decision is an important step. But the big “for-profit” publishers are not likely to go away without a fight. Real reform will require more than tweaking payment policies. It will require sustained coordination between libraries, funders, and academic institutions to reclaim ownership of the public good: science itself.

The prevailing view is that protecting research integrity, equity, and scientific quality requires flexibility, sustained investment in best practices, and structural reform of publishing markets. Blunt cost controls applied at the grant level may be too crude a remedy.

## Supplementary Material

qxag163_Supplementary_Data
