# Supplementary material for: Should NIH restrict the use of grant funds for publication costs? An analysis of public comments
Source: Health Aff Sch. 2026 Nov 5;4(11):qxag163. doi: 10.1093/haschl/qxag163 (PMC13738074; doi:10.1093/haschl/qxag163)
Supplement: Supplementary file 1 [file qxag163_supplementary_data.zip › qxag163_Supplementary_Data.docx]

ONLINE APPENDIX TABLES

## **Appendix Table1**

## **Illustrative Comments Expressing Concerns About APCs**

| **Commenter** | **Affiliation / Role** | **Illustrative Quote** | **Page** |
| --- | --- | --- | --- |
| Alex M | Organizational Official (academic) | “APCs… create inequalities as not everyone has funds to cover publishing fees… and have further consolidated the market in the big publishers.” | 37 |
| Anonymous commenter | Investigator (Academic institution) | “All proposed options risk shifting costs to authors or institutions, exacerbating inequities rather than resolving them.” | 40 |
| Anonymous commenter | Investigator (Academic institution) | “Capping allowable costs… is a blunt instrument that is likely to produce more harm than good.” | 40 |
| Kristin Riekert | Investigator/Researcher | “Publishers will capitalize on this requirement and increase their fees. This means more investigators will require even more funding year after year.” | 47 |
| Gail Bishop | University of Iowa; Investigator/Researcher | “Authors have increased publication costs, but no designated source of funds to pay for them.” | 33 |
| Anonymous commenter | Investigator (Non-profit research organization) | “Small labs cannot afford to publish in the most expensive journals.” | 35 |

# Appendix Table 2

# Selected Illustrative Comments on Peer Review, Reviewer Compensation, and Editorial Labor

| **Commenter** | **Affiliation / role** | **Illustrative quote** | **Page** |
| --- | --- | --- | --- |
| Gloria Greene | University of Alabama in Huntsville; organizational response | “Peer review is central to scientific integrity and needs proper resources … The ethical burden of asking underpaid academics to volunteer while industry profits from the system.” | 30 |
| Mary Cushman | University of Vermont; investigator | “No journals in my field compensate peer reviewers.” | 32 |
| Gail Bishop | University of Iowa; investigator and society journal editor | “Such non-profit entities could likely not afford to pay peer reviewers - this would put us at an even greater disadvantage than we already suffer compared to journals of large, for-profit commercial publishers.” | 33 |
| Dmitriy Sheyn | Cedars-Sinai Health Sciences University; investigator | “This [is] a great idea, because currently the peer review is huge burden on the reviewers, editors and investigators … Paid reviewers will be more diligent, time compliant and responsible.” | 36 |
| Alex M | Academic institution; organizational official | “The largest commercial publishers will be able to … outbid smaller, community-run journals for the best peer reviewers.” | 38 |
| Anonymous commenter | Academic institution; investigator | “NIH should treat grant peer review as serious, skilled labor—because it is.” | 40 |
| Philip Bayly | Washington University in St. Louis; investigator | “It is an activity that is currently framed as service to the field and scientific community. It requires a lot of time from highly skilled and well compensated people.” | 45 |
| Kristin Riekert | Investigator | “I have reviewed for >30 different journals … I have never been offered payment for my review.” | 47 |
| Gary L. Westbrook, MD | Vollum Institute; investigator | “There is no reason to pay for peer review as it is part of the academic enterprise. Paying will likely decrease the quality of reviews.” | 49 |
| Jodi Segal | Johns Hopkins; investigator and editor | “I have never been paid for peer review; I think it is just part of being a member of the scientific community.” | 50 |
| Aleksandar Rajkovic | UCSF; investigator | “Compensation for peer reviewers is a good idea … While potentially valuable, it may also inadvertently inflate APCs substantially beyond current norms.” | 51 |
| Cathy Stein, PhD | Case Western Reserve University; investigator | “I’ve never been paid to peer review a journal paper. I don’t know anyone that has, honestly.” | 53 |
| Richard K. Miller, PhD | University of Rochester; investigator | “For so many years, it has been an expectation of peer reviewers to assume the responsibilities for providing reviews. I disagree with the concept of paying for reviews.” | 58 |
| Cynthia Monaco | Investigator | “Peer reviewers aren’t compensated in general … I am concerned that requiring paid compensation for reviewers would further inflate the price of publication.” | 65 |

## Appendix Table 3

## Selected Public Comments Emphasizing the Importance of Peer Review

| **Theme** | **Subtheme** | **Commenter Name** | **Affiliation / Role** | **Verbatim Quote** |
| --- | --- | --- | --- | --- |
| Scientific integrity | Core function | Gloria Greene | Univ. of Alabama in Huntsville (organizational response) | “Peer review is central to scientific integrity and needs proper resources.” |
| Scientific rigor | Quality assurance | Wiley (publisher submission) | Commercial publisher | “Rigorous peer review contributes directly to reproducibility, unbiased evaluation, and the long-term trustworthiness of the scientific record.” |
| Public trust | Stewardship of taxpayer-funded science | Wiley (publisher submission) | Commercial publisher | “It also supports the public interest by helping ensure that research supported by taxpayer funds meets the highest possible standards before it is disseminated.” |
| Editorial process | Human judgment | Wiley (publisher submission) | Commercial publisher | “Editors and reviewers apply disciplinary context that automation cannot… rigorously evaluating manuscripts.” |
| System value | Labor intensity and expertise | Wiley (publisher submission) | Commercial publisher | “Each manuscript… requires significant human resources… involving… editors, peer reviewers, and subject-matter experts.” |
| System performance | Error prevention and reliability | Wiley (publisher submission) | Commercial publisher | “These processes prevent errors before publication… and support reproducibility.” |
| Reviewer function | Skilled labor | Anonymous commenter | Investigator | “NIH should treat grant peer review as serious, skilled labor—because it is.” |
| System sustainability | Reviewer burden reflects importance | Dmitriy Sheyn | Cedars-Sinai (investigator) | “Currently the peer review is [a] huge burden on the reviewers, editors and investigators.” |
| System improvement | Quality enhancement | Weifeng Gu | UC Riverside (investigator) | “Agree with compensation. This will help improve the journal quality.” |
| Community norm | Scientific service role | Richard K. Miller | Investigator | “Peer review… has historically been part of scientific service.” |
